# When negative customer engagement turns positive: A four-stage simulation model of engagement evolution in online brand communities

**DOI:** 10.1371/journal.pone.0360236

**Published:** 2026-11-11

**Authors:** Luning Zang, Yuying Liu, Linqiang Wang

**Affiliations:** 1 School of Management, Qufu Normal University, Rizhao, People's Republic of China; 2 School of Management, Shandong University, Jinan, People's Republic of China; 3 School of Tourism, Shandong Women’s University, Jinan, People's Republic of China; University of Naples Federico II: Universita degli Studi di Napoli Federico II, ITALY

## Abstract

This study investigates whether and when service recovery can convert negative customer engagement (NCE) into positive customer engagement (PCE) in online brand communities, and how early value conditions shape later engagement trajectories. Using Xiaomi Community as the focal context, we develop a four-stage threshold-based simulation framework in which consumer utility is treated as a latent evaluation antecedent rather than customer engagement itself. An explicit non-engagement (NE) interval is positioned between the negative and positive thresholds so that moderately evaluated users are not forced into PCE or NCE. Price, product quality, service quality, and personal needs met are retained as the focal antecedents. Core scenarios are repeated 1,000 times with recorded random seeds and 95% simulation uncertainty intervals, and robustness is examined across conversion rates, thresholds, initial conditions, coefficient distributions, sign-allocation rules, and simulation horizons. Price and product quality show strong and opposite effects on the PCE-NCE balance, and a direct price-by-quality analysis indicates that higher quality attenuates the adverse effect of higher price. Service-quality effects remain under both Stage-4-only and continuous-service architectures, but the results do not support a general claim that later-stage service investment is superior to earlier service. Targeted recovery applied to NCE users produces a stronger and more stable NCE-to-PCE repair effect. The results clarify the assumptions, robustness, and managerial boundary conditions of the stage-based model. Because coefficient sign allocation remains a major structural uncertainty, the reported quantities are interpreted as scenario outputs and robust directional comparisons rather than forecasts of actual PCE, NCE, or NE population shares.

## 1. Introduction

Online brand communities (OBCs) are arenas where users share experiences and interact with brands. The present study focuses specifically on brand-directed customer engagement behavior expressed within Xiaomi's online brand community. User-generated content can produce two customer engagement (CE) behaviors [1,2]: positive customer engagement (PCE), expressed through support and recommendations, and negative customer engagement (NCE), expressed through complaints and negative word of mouth. Customer engagement concerns customers’ cognitive, affective, and behavioral investment in a focal brand, whereas online brand community engagement additionally captures motives for participating in the community itself [3–6]. These behaviors vary over time and may convert as firms adjust strategies and users reassess experiences [7]. Importantly, not every moderately satisfied or dissatisfied customer manifests an observable positive or negative behavior; some users may remain non-engaged (NE).

The Xiaomi setting is particularly relevant because smartphone communities combine product experience, software updates, price evaluations, service encounters, and peer interaction. Research using Xiaomi Community and Huawei Fan Club users shows that online service brand communities provide an important setting for customer brand engagement [8]. Evidence from Samsung's online brand community during a product-harm crisis likewise illustrates how product problems and recovery responses can reshape community engagement and subsequent brand responses [9]. These technology-oriented OBC studies provide a closer category match than examples drawn from unrelated community settings and help anchor the present four-stage model in the focal context.

These observations suggest that price, product quality, service quality, and personal needs met level jointly shape CE formation and evolution. Their effects are dynamic rather than linear. Price changes recalibrate value assessments and switching intentions, while product quality affects continued use and content co-creation. Service quality shapes post-purchase evaluations, and service recovery refers specifically to corrective action following a service failure or unresolved customer problem [10–13]. Personal needs met captures the fit between brand offerings and situational requirements. Accordingly, this study examines how these determinants drive stage-specific differentiation and transformation between PCE and NCE, informing governance strategies that promote PCE and curb NCE. In the model, these determinants are treated as antecedent evaluations rather than as CE itself, and numerical settings that are not directly calibrated are treated as normalized or structural assumptions and sensitivity-tested.

## 2. Literature review

CE is commonly conceptualized as a multidimensional construct that integrates interaction-based cognitive and affective states with motivation-driven behaviors that extend beyond transactions. Within retailing, CE is increasingly linked to relationship building across digitally mediated touchpoints, where customers’ appraisals and interactions shape downstream responses from both customers and firms. This framing is particularly salient in online brand communities (OBCs), where engagement is observable through user-generated content and where the ongoing customer journey can be traced through recurring interactions rather than isolated actions. Because the present study observes brand-directed expressions within an online community, CE denotes engagement with the focal brand, whereas the OBC is treated as the interaction context; motives for participating in the community itself are acknowledged as related but conceptually distinct [3–7].

A growing stream further recognizes that engagement is not uniformly positive. In OBCs, positive and negative customer engagement can co-exist and shift as experiences, expectations, and interpretations change. Behavioral regularities and disruptions in consumer decision contexts also suggest that engagement trajectories can be sensitive to perceived progress and setbacks, reinforcing the plausibility of directional shifts and reversals over time. Moreover, the logics underlying positive and negative expressions are not fully symmetric. Negative engagement is more likely to be associated with critical incidents and intensified emotional and coping responses, consistent with mechanisms that emphasize appraisal processes and the role of affect. Taken together, prior work motivates treating PCE and NCE as jointly modeled outcomes whose balance may change with the unfolding interaction experience in OBCs. The model additionally defines non-engagement (NE) as non-manifestation when latent evaluation falls within the central threshold interval; NE is not forced into either PCE or NCE.

The four antecedents are retained for distinct theoretical reasons. Price and product quality enter early evaluation through perceived-value logic: price represents sacrifice and product quality represents benefits received, so their balance shapes favorable or unfavorable appraisal [10]. Service quality is a post-purchase evaluation cue [11], whereas service recovery is a corrective response to failure or an unresolved customer problem and can reshape subsequent customer responses [12,13]. Personal needs met represents the fit between the brand offering and customers’ motives or situational requirements, consistent with engagement research linking antecedent appraisals and motivations to subsequent engagement responses [14–16]. These sources justify the conceptual inclusion and expected directions of the variables; they do not provide transferable numerical thresholds, so the model's numerical settings are treated as normalized assumptions and sensitivity-tested.

Importantly, CE is increasingly viewed as a dynamic process that evolves through repeated interaction and feedback rather than a stable state captured by single indicators. Recent research highlights that engagement intensity can fluctuate, that behaviors can feed back into future propensities, and that engagement is embedded in multi-actor environments where peers and norms shape trajectories. Interactivity can stimulate participation, yet it can also generate withdrawal and negative word of mouth through social influence, suggesting that CE dynamics operate across individual, network, and contextual levels. Nonlinear patterns further complicate intervention: contexts involving identity, exclusion, or heightened social meaning can produce backlash when responsiveness or targeting is perceived as misaligned, implying threshold-like changes rather than smooth responses. Methodologically, these dynamics have encouraged process-sensitive approaches, including behavioral measurement using high-frequency traces and text-based evidence, as well as computational approaches for modeling customer engagement patterns [7,17–19]. The four-stage ordering used here is therefore treated as an analytical customer-journey sequence rather than an empirically universal hierarchy. It reflects when information becomes salient in the model—price at purchase, product quality through use, and service or recovery through post-purchase encounters—while personal-needs fulfillment is placed in Stage 4 as a simplifying assumption. The most consequential service-timing assumption is tested through an alternative continuous-service architecture, and the possibility that personal needs also operate earlier is retained as a limitation.

Overall, the literature implies that explaining PCE and NCE, and especially their conversion, requires models that represent stage-based change, feedback, and potential turning points. Accordingly, this study adopts a dynamic lens to examine how price, product quality, service quality, and personal needs met shape the evolution and transformation of PCE and NCE in OBCs. Within the four-stage structure, Stage 1 represents purchase/entry, Stage 2 potential engagement orientation, Stage 3 behavioral manifestation, and Stage 4 later updating/recovery. The model incorporates an explicit NE middle state and targeted recovery while retaining the threshold-based architecture.

## 3. Materials and models

Existing research conceptualizes CE behavior as a dynamic process triggered by antecedents and generated through cognitive appraisal and affective processing. Building on this view, we develop a multi-stage mathematical model and represent the formation of PCE and NCE, as well as their mutual transformation, in four stages. In Stage 1, consumers purchase and enter the initial customer population. In Stage 2, customers form engagement intention from usage experiences and community observation and move into a potential engagement state, because expressing CE requires resources (time, effort, knowledge). Potential PCE and NCE are differentiated mainly by utility differences driven by price and product quality. In Stage 3, as experiences deepen, a subset of potential users externalizes engagement as observable PCE or NCE, with stage-varying sensitivity to price, product quality, and service quality. In Stage 4, product issues and after-sales interactions, together with community-based support and demand expression, generate heterogeneous service quality perceptions that induce bidirectional conversion between PCE and NCE and sustain their evolution. To represent the middle state while retaining the four-stage architecture, the model includes an explicit non-engagement (NE) interval, interprets Utility(i,t) as a latent evaluation antecedent rather than CE itself, and distinguishes routine service quality from targeted service recovery. Fig 1 provides a plain-language overview of the analytical workflow.

**Fig 1:**
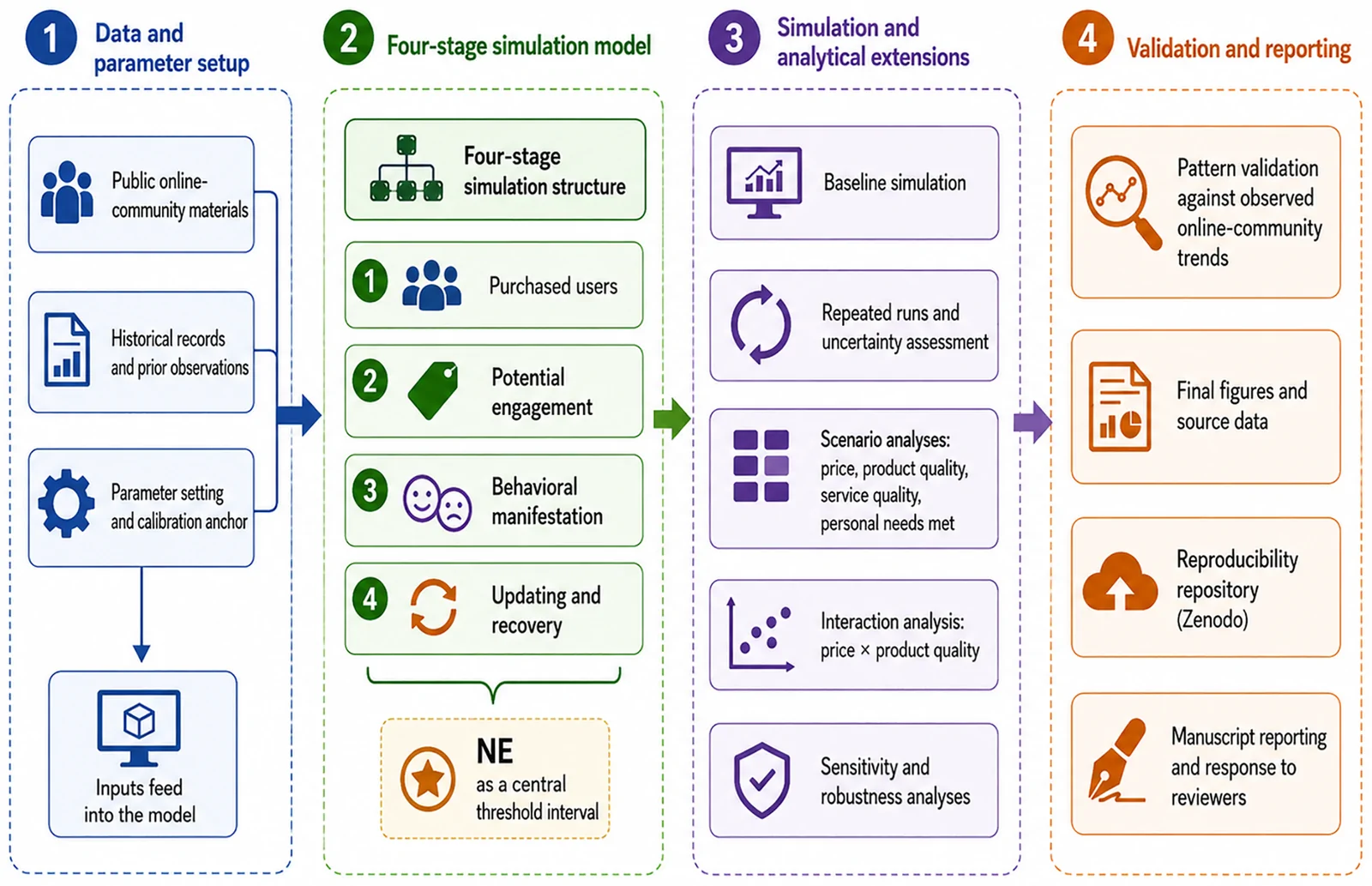
Analytical workflow of the study. The workflow summarizes four connected phases: data and parameter setup; four-stage simulation model construction; simulation and analytical extensions; and validation and reporting.

For readers new to simulation methods, the analytical workflow is presented separately from the conceptual state-transition diagram. Fig 1 organizes the study into four linked phases: data and parameter setup; construction of the four-stage simulation model; baseline, repeated-run, scenario, interaction, sensitivity and robustness analyses; and pattern validation, reproducibility, and reporting.

### 3.1 Model assumptions

In Stage 1, users purchase the product and become purchased users. The stage-based increment in purchased users follows a Sigmoid function, capturing rapid early growth after launch and subsequent stabilization. In Stage 2, purchased users enter the potential engagement state at a fixed conversion rate, so the potential engagement scale grows proportionally with the purchased user base. For tractability, we assume equal stage duration (T = 1) and use the threshold logic with a central NE interval: utility below the negative threshold indicates potential NCE, utility above the positive threshold indicates potential PCE, and utility between the two thresholds is retained as NE.

Utility is determined by price, product quality, service quality, and personal needs met. Price dissatisfaction increases the likelihood of NCE. Higher product quality and service quality promote PCE and may facilitate conversion from NCE to PCE, while deterioration has the opposite effect. Personal needs met promotes PCE, but unmet needs do not necessarily induce NCE or trigger conversion from PCE to NCE. Within the threshold-based framework, we use a linear utility specification and bounded sensitivities: price in [−1, 0], product quality and service quality in [−1, 1], and personal needs met in [0, 1]. These numerical bounds are model assumptions rather than values estimated from the cited CE literature. To capture heterogeneity and dynamics, these sensitivity coefficients are allowed to vary across stages. For conceptual clarity, Utility(i,t) is interpreted as a latent evaluation antecedent rather than an operationalization of CE itself. Targeted service recovery is not assigned a new core coefficient; in Stage 4 it is represented as a user-specific improvement in the service-quality input for eligible NCE users.

### 3.2 Model establishment

Based on the four stage partition and the associated assumptions, we model the evolutionary process of CE in a stage specific manner.

#### 3.2.1 Stage 1.

In Stage 1, after product launch, a share of market users purchase based on perceived value and enter the purchased-user pool. Consistent with the assumptions, stage-wise growth in purchased users follows a Sigmoid function. Let the number of purchased users in Stage 1 be Base_Add. The newly converted purchased users per stage, Number_of_TotalAdd(t), are expressed as:


$$\begin{array}{c} \text{Number}\_\text{of}\_\text{TotalAdd}(\text{t})\;=\;\text{Base}\_\text{Add}\;\times \;\frac{\text{1}}{\text{1}+e^{-t}} \end{array}$$ (1)

t denotes time.

#### 3.2.2 Stage 2.

Purchased users from Stage 1 transition to the potential engagement state in Stage 2. The conversion rate is denoted Rate_of_PotentialCE and is assumed to be fixed. Users who do not convert remain in the purchased state and are carried over to the next stage, accumulating in the purchased-user population (dashed arrow in Fig 2). Thus, at time t, the total purchased users Number_of_Total(t) and the newly converted potential engagement users Number_of_PotentialCE_Add(t) are given by. NE is introduced only at the subsequent potential-engagement classification step.

**Fig 2:**
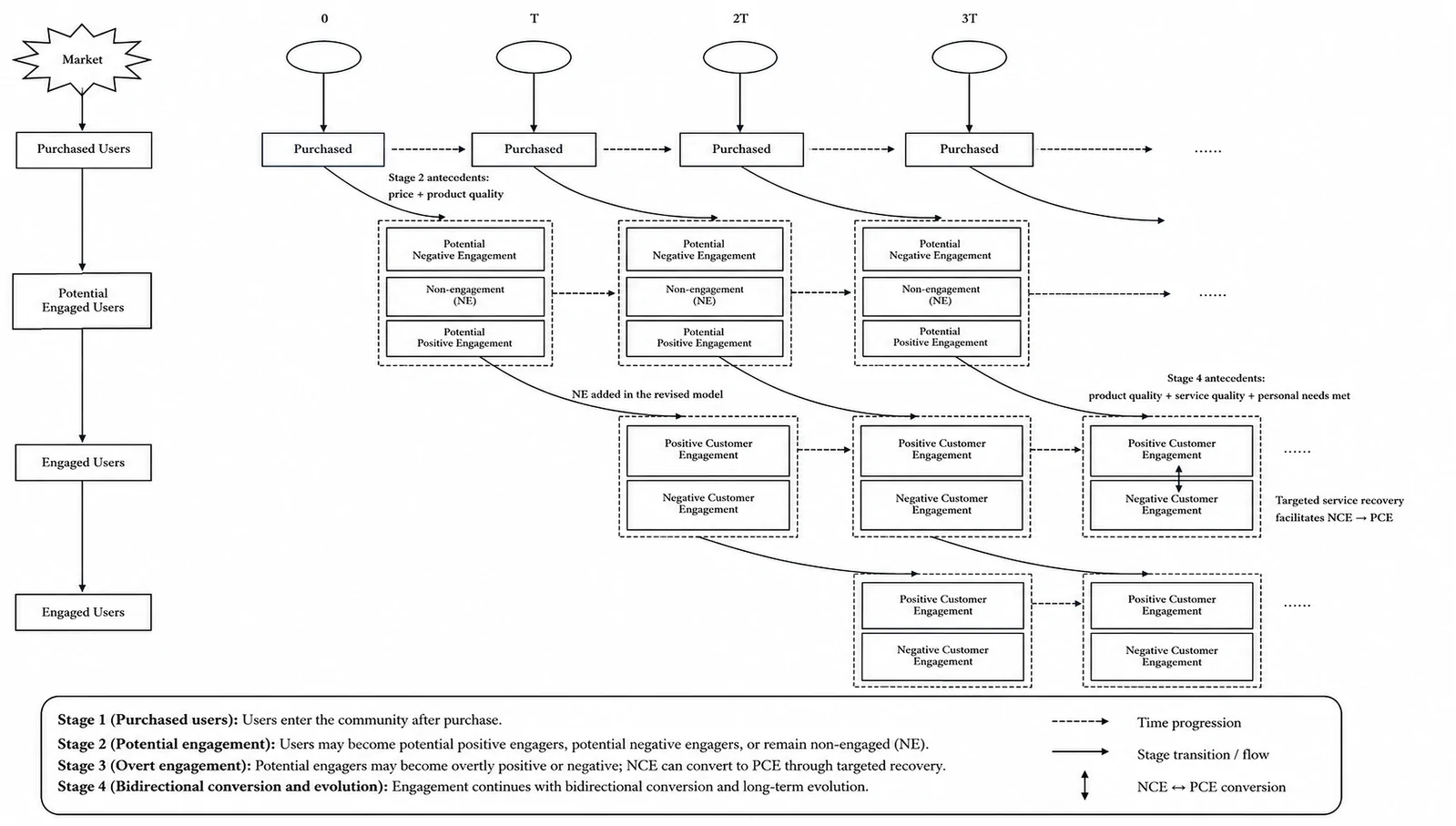
Four-stage evolution of customer engagement behaviors. The four-stage architecture links purchase and entry, potential engagement, behavioral manifestation, and later updating/recovery. An explicit NE interval lies between the negative and positive thresholds; Utility(i,t) is treated as a latent evaluation antecedent rather than CE itself; and targeted service recovery is distinguished from routine service quality.


$$\begin{array}{c} \text{Number}\_\text{of}\_\text{Total}(\text{t})\;=\;\left\{ \begin{array}{c} \text{Number}\_\text{of}\_\text{TotalAdd}(\text{0})\;,\text{ t}=\text{0}\\ \text{Number}\_\text{of}\_\text{TotalAdd}(\text{t})\\ +\;\text{Number}\_\text{of}\_\text{Total}(\text{t}-\text{1})\\ \times \;(\text{1}\;-\;\text{Rate}\_\text{of}\_\text{PotentialCE})\;,\text{ t}>\text{0} \end{array}\right. \end{array}$$ (2)

$$\begin{array}{c} \text{Number}\_\text{of}\_\text{PotentialCE}\_\text{Add}(\text{t})\;=\;\left\{ \begin{array}{c} \text{0}\;,\text{ t}=\text{0}\\ \text{Number}\_\text{of}\_\text{Total}(\text{t})\;\times \;\text{Rate}\_\text{of}\_\text{PotentialCE },\text{ t}>\text{0} \end{array}\right. \end{array}$$ (3)

Potential engagement users are further classified as potential PCE or potential NCE by comparing consumer utility with the potential engagement threshold. Let Utility(i, t) denote the utility of user i at time t. The initial product price is Initial_of_Price, the initial product quality level is Initial_Quality_of_Production, the initial service quality level is Initial_Quality_of_Service, and user personal needs are Initial_of_PersonalNeeds. The associated sensitivity coefficients are α, β, δ, and γ. We draw each coefficient magnitude independently from a Uniform(0, 1) distribution and assign signs in a separate step, yielding:


$$\begin{array}{c} \begin{array}{c} \text{Utility}(\text{i},\text{t})\;=\;\alpha \;\times \;\text{Initial}\_\text{of}\_\text{Price}\;+\;\beta \;\times \;\text{Initial}\_\text{Quality}\_\text{of}\_\text{Production}\\ +\;\delta \;\times \;\text{Initial}\_\text{Quality}\_\text{of}\_\text{Service}\;+\;\gamma \;\times \;\text{Initial}\_\text{of}\_\text{PersonalNeeds} \end{array} \end{array}$$ (4)

where α∈[−1,0], β∈[−1,1], δ∈[−1,1], and γ∈[0,1]. Improving Initial_Quality_of_Production and Initial_Quality_of_Service increases utility on average; however, for users in the potential NCE state or the NCE state, β and δ may be negative. To reflect the idea that quality improvement reduces adverse perceptions, we constrain the sign distribution of β and δ such that the probability of negative values decreases as quality increases, while the probability of positive values rises. This rule is implemented via Assignment_Perception_Coefficient. More explicitly, Uniform(0,1) applies to coefficient magnitudes; α always receives a negative sign and γ remains non-negative. For the reference sign-allocation rule inherited from Assignment_Perception_Coefficient, the probability of a negative product-quality coefficient is Pr(β < 0)=min(1,1/Q), and the probability of a negative service-quality coefficient is Pr(δ < 0)=1/(S+1), where Q and S are the applicable normalized product- and service-quality inputs. At Q = 2 and S=1, both negative-sign probabilities equal 0.50. These are algorithmic structural rules rather than empirically estimated probabilities; fixed 0.25, 0.50, 0.75 and asymmetric sign-probability alternatives, together with alternative magnitude distributions, are therefore examined in sensitivity analysis.

In this stage, users only experience perceived product value and product quality, so Initial_Quality_of_Service = 0 and Initial_of_PersonalNeeds = 0 in Utility(i, t). Let ThresholdValue_of_PotentialNCE denote the utility threshold for conversion from the purchased state to the potential NCE state, and let Number_of_PotentialNCEAdd(t) denote the number of users making this conversion at time t. Similarly, ThresholdValue_of_PotentialPCE is the threshold for conversion to the potential PCE state, and Number_of_PotentialPCEAdd(t) denotes the corresponding number of users at time t. To incorporate the middle state without changing the underlying threshold mechanism, an explicit Number_of_PotentialNEAdd(t) is inserted between the potential NCE and potential PCE equations. Users whose Utility(i,t) falls within the central interval are counted as NE; the interval width is varied in sensitivity analysis.


$$\begin{array}{c} \text{Number}\_\text{of}\_\text{PotentialNCEAdd}(\text{t})\;=\;\{\sum_{\begin{array}{c} \text{i}\in \text{Number}\_\text{of}\_\text{PotentialCE}(\text{t}-\text{1})\\ \text{Utility}(\text{i},\text{t})<\text{ThresholdValue}\_\text{of}\_\text{PotentialNCE} \end{array}}^{\text{0}\;,\text{ t}=\text{0}}i,\text{ t}>\text{0} \end{array}$$ (5)

$$\begin{array}{c} \text{Number}\_\text{of}\_\text{PotentialNEAdd}(\text{t})\;=\;\{\sum_{\begin{array}{c} \text{i}\in \text{Number}\_\text{of}\_\text{PotentialCE}(\text{t}-\text{1})\\ \text{ThresholdValue}\_\text{of}\_\text{PotentialNCE}\leq \text{Utility}(\text{i},\text{t})\\ \text{Utility}(\text{i},\text{t})\leq \text{ThresholdValue}\_\text{of}\_\text{PotentialPCE} \end{array}}^{\text{0}\;,\text{ t}=\text{0}}i\;,\text{ t}>\text{0} \end{array}$$ (6)

$$\begin{array}{c} \text{Number}\_\text{of}\_\text{PotentialPCEAdd}(\text{t})\;=\;\{\sum_{\begin{array}{c} \text{i}\in \text{Number}\_\text{of}\_\text{PotentialCE}(\text{t}-\text{1})\\ \text{Utility}(\text{i},\text{t})>\text{ThresholdValue}\_\text{of}\_\text{PotentialPCE} \end{array}}^{\text{0}\;,\text{ t}=\text{0}}i,\text{ t}>\text{0} \end{array}$$ (7)

#### 3.2.3 Stage 3.

Users in the potential CE state in Stage 2 transition to the CE state in Stage 3, while nonconverting users are carried over and accumulate in the next stage. Conversion is determined by comparing consumer utility with the CE-state threshold. Let ThresholdValue_NCE and ThresholdValue_PCE denote the thresholds for conversion from potential NCE to NCE and from potential PCE to PCE. At time t, Number_of_NCEAdd_Third(t) and Number_of_PCEAdd_Third(t) denote the numbers converting from potential NCE and potential PCE, respectively. Let Number_of_Potential_NCE(t) and Number_of_Potential_PCE(t) denote the sizes of the potential NCE and potential PCE populations. After Stage 3, the resulting state sizes are Number_of_NCE_Third(t) and Number_of_PCE_Third(t), respectively, as follows: Potential NE users are carried forward as non-engaged in the core specification; this adds the middle state without introducing additional re-entry parameters that cannot be independently calibrated.


$$\begin{array}{c} \text{Number}\_\text{of}\_\text{NCEAdd}\_\text{Third}(\text{t})\;=\;\left\{ \begin{array}{c} \text{0}\;,\text{ t}=\text{0},\text{1}\\ \sum_{\begin{array}{c} \text{i}\in \text{Number}\_\text{of}\_\text{PotentialNCE}(\text{t}-\text{1})\\ \text{Utility}(\text{i},\text{t})<\text{ThresholdValue}\_\text{NCE} \end{array}}i\;,\text{ t}>\text{1} \end{array}\right. \end{array}$$ (8)

$$\begin{array}{c} \text{Number}\_\text{of}\_\text{PCEAdd}\_\text{Third}(\text{t})\;=\;\left\{ \begin{array}{c} \text{0}\;,\text{ t}=\text{0},\text{1}\\ \sum_{\begin{array}{c} \text{i}\in \text{Number}\_\text{of}\_\text{PotentialPCE}(\text{t}-\text{1})\\ \text{Utility}(\text{i},\text{t})>\text{ThresholdValue}\_\text{PCE} \end{array}}i\;,\text{ t}>\text{1} \end{array}\right. \end{array}$$ (9)

$$\begin{array}{c} \text{Number}\_\text{of}\_\text{PotentialNCE}(\text{t})\;=\;\left\{ \begin{array}{c} \text{0}\;,\text{ t}=\text{0}\\ \text{Number}\_\text{of}\_\text{PotentialNCEAdd}(\text{t})\;,\text{ t}=\text{1}\\ \text{Number}\_\text{of}\_\text{PotentialNCEAdd}(\text{t})\\ +\;\text{Number}\_\text{of}\_\text{PotentialNCE}(\text{t}-\text{1})\\ -\;\text{Number}\_\text{of}\_\text{NCEAdd}\_\text{Third}(\text{t})\;,\text{ t}>\text{1} \end{array}\right. \end{array}$$ (10)

$$\begin{array}{c} \text{Number}\_\text{of}\_\text{PotentialNE}(\text{t})\;=\;\left\{ \begin{array}{c} \text{0}\;,\text{ t}=\text{0}\\ \text{Number}\_\text{of}\_\text{PotentialNEAdd}(\text{t})\;,\text{ t}=\text{1}\\ \text{Number}\_\text{of}\_\text{PotentialNEAdd}(\text{t})\\ +\;\text{Number}\_\text{of}\_\text{PotentialNE}(\text{t}-\text{1})\;,\text{ t}>\text{1} \end{array}\right. \end{array}$$ (11)

$$\begin{array}{c} \text{Number}\_\text{of}\_\text{PotentialPCE}(\text{t})\;=\;\left\{ \begin{array}{c} \text{0}\;,\text{ t}=\text{0}\\ \text{Number}\_\text{of}\_\text{PotentialPCEAdd}(\text{t})\;,\text{ t}=\text{1}\\ \text{Number}\_\text{of}\_\text{PotentialPCEAdd}(\text{t})\\ +\;\text{Number}\_\text{of}\_\text{PotentialPCE}(\text{t}-\text{1})\\ -\;\text{Number}\_\text{of}\_\text{PCEAdd}\_\text{Third}(\text{t})\;,\text{ t}>\text{1} \end{array}\right. \end{array}$$ (12)

$$\begin{array}{c} \text{Number}\_\text{of}\_\text{NCE}\_\text{Third}(\text{t})\;=\;\sum_{\text{t1}=\text{0}}^{\text{t}}\text{Number}\_\text{of}\_\text{NCEAdd}\_\text{Third}(\text{t1}) \end{array}$$ (13)

$$\begin{array}{c} \text{Number}\_\text{of}\_\text{PCE}\_\text{Third}(\text{t})\;=\;\sum_{\text{t1}=\text{0}}^{\text{t}}\text{Number}\_\text{of}\_\text{PCEAdd}\_\text{Third}(\text{t1}) \end{array}$$ (14)

#### 3.2.4 Stage 4.

In the reference architecture, users who formed PCE and NCE in Stage 3 update their evaluations in Stage 4 as product and service experiences deepen. Service quality and personal needs met become operative at this stage by model construction; this timing is an analytical assumption rather than a universal empirical claim. Accordingly, in the consumer utility function for this stage, Initial_Quality_of_Service and Initial_of_PersonalNeeds are no longer set to 0. Let Number_of_Transfer_to_NCE(t) and Number_of_Transfer_to_PCE(t) denote the numbers converting from PCE to NCE and from NCE to PCE in this stage. Let Number_of_NCEAdd_Forth(t) and Number_of_PCEAdd_Forth(t) denote the numbers carried over from the previous time point in the NCE and PCE states. After Stage 4 conversions, the accumulated totals at time t are Number_of_NCE_Forth(t) and Number_of_PCE_Forth(t), respectively. For the targeted-recovery test, service recovery is represented only by increasing the Stage-4 service-quality input for eligible NCE users; no new recovery coefficient is added. A continuous-service alternative, in which routine service quality is active before Stage 4, is analyzed separately to test whether the later-stage result is induced by the stage-specific architecture.

All recorded states are end-of-period stocks. Period t begins from the stocks recorded at t-1. Equations (15)-(16) evaluate eligibility for PCE-to-NCE and NCE-to-PCE transfer from these opening realized stocks, but the resulting transfer counts are period-t flows and are applied during the same period t. Equations (17)-(20) therefore index the eligible opening stock by t-1 and the applied transfer by t; current Stage-3 manifestations are added afterward to form the end-of-period realized stocks.


$$\begin{array}{c} \text{Number}\_\text{of}\_\text{Transfer}\_\text{to}\_\text{NCE}(\text{t})\;=\;\{\sum_{\begin{array}{c} \text{i}\in \text{Number}\_\text{of}\_\text{PCE}\_\text{Forth}(\text{t}-\text{1})\\ \text{Utility}(\text{i},\text{t})<\text{ThresholdValue}\_\text{NCE} \end{array}}^{\text{0}\;,\text{ t}=\text{0},\text{1},\text{2}}i,\text{ t}>\text{2} \end{array}$$ (15)

$$\begin{array}{c} \text{Number}\_\text{of}\_\text{Transfer}\_\text{to}\_\text{PCE}(\text{t})\;=\;\{\sum_{\begin{array}{c} \text{i}\in \text{Number}\_\text{of}\_\text{NCE}\_\text{Forth}(\text{t}-\text{1})\\ \text{Utility}(\text{i},\text{t})>\text{ThresholdValue}\_\text{PCE} \end{array}}^{\text{0}\;,\text{ t}=\text{0},\text{1},\text{2}}i\;,\text{ t}>\text{2} \end{array}$$ (16)

$$\begin{array}{c} \text{Number}\_\text{of}\_\text{NCEAdd}\_\text{Forth}(\text{t})\;=\;\left\{ \begin{array}{c} \text{Number}\_\text{of}\_\text{NCE}\_\text{Third}(\text{t})\;,\text{ t}=\text{0},\text{1},\text{2}\\ \text{Number}\_\text{of}\_\text{NCE}\_\text{Forth}(\text{t}-\text{1})\\ -\;\text{Number}\_\text{of}\_\text{Transfer}\_\text{to}\_\text{PCE}(\text{t})\\ +\;\text{Number}\_\text{of}\_\text{Transfer}\_\text{to}\_\text{NCE}(\text{t})\;,\text{ t}>\text{2} \end{array}\right. \end{array}$$ (17)

$$\begin{array}{c} \text{Number}\_\text{of}\_\text{PCEAdd}\_\text{Forth}(\text{t})\;=\;\left\{ \begin{array}{c} \text{Number}\_\text{of}\_\text{PCE}\_\text{Third}(\text{t})\;,\text{ t}=\text{0},\text{1},\text{2}\\ \text{Number}\_\text{of}\_\text{PCE}\_\text{Forth}(\text{t}-\text{1})\\ -\;\text{Number}\_\text{of}\_\text{Transfer}\_\text{to}\_\text{NCE}(\text{t})\\ +\;\text{Number}\_\text{of}\_\text{Transfer}\_\text{to}\_\text{PCE}(\text{t})\;,\text{ t}>\text{2} \end{array}\right. \end{array}$$ (18)

$$\begin{array}{c} \text{Number}\_\text{of}\_\text{NCE}\_\text{Forth}(\text{t})\;=\;\left\{ \begin{array}{c} \text{Number}\_\text{of}\_\text{NCEAdd}\_\text{Forth}(\text{t})\;,\text{ t}=\text{0},\text{1},\text{2}\\ \text{Number}\_\text{of}\_\text{NCEAdd}\_\text{Forth}(\text{t})\\ +\;\text{Number}\_\text{of}\_\text{NCEAdd}\_\text{Third}(\text{t})\;,\text{ t}>\text{2} \end{array}\right. \end{array}$$ (19)

$$\begin{array}{c} \text{Number}\_\text{of}\_\text{PCE}\_\text{Forth}(\text{t})\;=\;\left\{ \begin{array}{c} \text{Number}\_\text{of}\_\text{PCEAdd}\_\text{Forth}(\text{t})\;,\text{ t}=\text{0},\text{1},\text{2}\\ \text{Number}\_\text{of}\_\text{PCEAdd}\_\text{Forth}(\text{t})\\ +\;\text{Number}\_\text{of}\_\text{PCEAdd}\_\text{Third}(\text{t})\;,\text{ t}>\text{2} \end{array}\right. \end{array}$$ (20)

Population conservation follows from six mutually exclusive and exhaustive end-of-period stocks. Let C(t) denote all users cumulatively entering the model by period t:


$$\begin{array}{c} C(t)=Purchased(t)+PotentialNCE(t)+NE(t)+PotentialPCE(t)+NCE(t)+PCE(t). \end{array}$$ (21)

A Stage-3 manifestation removes one user from a potential stock and adds the same user to the corresponding realized stock. A Stage-4 transfer removes one user from one realized stock and adds that user to the other. Each transition therefore has zero net population effect, no user occupies more than one end-of-period stock, and the implementation checks Equation (21) and non-negativity at every period.

### 3.3 Model algorithm

Based on the CE evolutionary rules above, we implement the proposed four-stage process via a simulation algorithm (Table 1). Inputs include total evolution steps, base growth of purchased users, the conversion rate from purchased to potential CE, and initial parameter settings (price, product quality, service quality, and personal needs met). At each time step, the algorithm updates purchased users using sigmoid growth with retention, then generates potential CE users at a fixed conversion rate. It computes user utility under the current configuration and assigns new potential users to potential NCE, NE, or potential PCE using threshold rules. Potential users may then convert into NCE or PCE, and in the later stage, bidirectional transfers between NCE and PCE occur based on transfer thresholds. Iterating these steps yields time-series outputs of key state variables, enabling assessment of CE trajectories and comparison of behavioral patterns across parameter settings. The algorithm records random seeds for repeated runs, documents the sign-allocation rule, applies targeted recovery only to eligible NCE users, and checks non-negative states and population conservation. In particular, Stage-4 transfers are evaluated from opening realized stocks and applied in the same period before current Stage-3 manifestations are added.

**Table 1:** Model algorithm.

Input:	
	Total steps T; Base_Add; Rate_of_PotentialCE; Price; ProductQuality; ServiceQuality; PersonalNeeds; potential, manifestation, and transfer thresholds; service-timing architecture; recovery increment; random seed.
Output:	
	Time-series records for purchased users, PotentialNCE, NE, PotentialPCE, realized NCE/PCE, and Stage-4 transfer counts.
1:	Initialize state variables and the recorded random seed.
2:	for each time step t in 0..T do
3:	Compute new purchased-user additions using Equation (1).
4:	Update the purchased-user stock using Equation (2).
5:	Compute new potential-engagement additions using the fixed Rate_of_PotentialCE, Equation (3).
6:	Compute the latent evaluation Utility(i,t) using Equation (4).
7:	Assign new potential users to PotentialNCE, NE, or PotentialPCE using Equations (5)-(7).
8:	if t is in the potential-to-CE manifestation period then
9:	Compute PotentialNCE users manifesting NCE using Equation (8).
10:	Compute PotentialPCE users manifesting PCE using Equation (9).
11:	Update PotentialNCE, NE, and PotentialPCE stocks using Equations (10)-(12).
12:	Update cumulative realized NCE and PCE using Equations (13)-(14).
13:	else retain potential stocks by accumulation.
14:	end if
15:	if t is in the Stage-4 transfer period then
16:	Compute PCE-to-NCE transfers using Equation (15).
17:	Compute NCE-to-PCE transfers using Equation (16). For targeted recovery scenarios, increase the service-quality input only for eligible NCE users.
18:	Apply period-t transfers to the opening realized stocks in the same period, then add current Stage-3 manifestations to obtain end-of-period NCE and PCE using Equations (17)-(20).
19:	else keep transfer counts at zero.
20:	end if
21:	Check non-negative states and exact population conservation using Equation (21).
22:	Record state and transition variables at period t.
23:	end for
24:	return time-series records and terminal metrics.

Note. T denotes the total number of simulation periods. Base_Add denotes the baseline number of newly added purchased users. PotentialCE denotes the potential-engagement pool. PotentialNCE and PotentialPCE denote potential negative and positive customer engagement; NE denotes non-engagement. NCE and PCE denote realized negative and positive customer engagement. The algorithm uses the four-stage threshold logic with a neutral interval, repeated-run uncertainty analysis, explicit sign-allocation documentation, and service-timing robustness checks.

### 3.4 Ethics and data protection

This retrospective, non-interventional observational study was reviewed and approved by the Biomedical Ethics Committee of Qufu Normal University (Ethics Approval No. 2026170; August 6, 2026). The study analyzes pre-existing, publicly accessible online-community materials and author-generated simulation data; no individuals were recruited, contacted, or experimentally intervened upon. The Committee waived the requirement for individual informed consent because the study involved retrospective analysis of publicly accessible materials, no direct interaction with users, and no more than minimal risk to individuals. In accordance with the approved data-sharing conditions, author-generated simulation code, parameter files, recorded random seeds, simulation outputs, figure source data, and de-identified or aggregated validation results may be shared publicly. Raw online-community posts, usernames, user IDs, persistent platform identifiers, verbatim user-generated text, precise timestamps, and other information that could facilitate re-identification are not publicly disclosed.

## 4. Simulation results

To further examine the dynamic evolution of positive customer engagement and negative customer engagement in online brand communities, this section conducts a simulation analysis based on the proposed four-stage model. The simulation aims to illustrate how purchased users gradually transform into potential engagement users, overt engagement users, and mutually convertible PCE and NCE users over time. It also enables us to explore how key managerial factors, including price, product quality, service quality, and personal needs met, affect the formation and transformation of different engagement behaviors. Through this simulation, the theoretical mechanism proposed in the previous sections can be translated into observable dynamic patterns. To quantify stochastic uncertainty and reproducibility, scenario trajectories are supplemented with seeded repeated runs and simulation uncertainty intervals while retaining the same scenario sequence.

### 4.1 Simulation setting

For Rate_of_PotentialCE, we use a descriptive ratio based on the 198,000 members of the Mi 11 Ultra circle in Xiaomi Community and the corresponding sales quantity over 20.5 months: 19.8/(9.44 x 20.5) = 0.10. Community membership, product purchase, and potential engagement are related but non-equivalent quantities. This calculation therefore does not calibrate a behavioral conversion probability. The value 0.10 is used only as a proxy-based scenario anchor for the reference configuration, and 0.05, 0.10, and 0.20 are examined in sensitivity analysis.


$$\begin{array}{c} \text{Rate}\_\text{of}\_\text{PotentialCE}\;=\;\frac{\text{1}\text{9}\text{8}\text{0}\text{0}\text{0}}{\text{9}\text{4}\text{4}\text{0}\text{0}\times \text{2}\text{0}.\text{5}}\;=\;\text{0}.\text{1}\text{0} \end{array}$$

Published CE and adjacent service/retailing studies support the directional relevance of price and perceived value, product quality, service quality and recovery, and customer motives, but they operationalize these constructs on study-specific empirical scales rather than provide transferable transition probabilities or thresholds for this simulation [10–16]. We therefore do not import their numerical scales into the model. The reference values below are normalized scenario settings or structural assumptions, and their plausibility is evaluated through systematic sensitivity analysis rather than presented as empirically estimated CE parameters.

Given the limited CE-focused simulation evidence and the need for subsequent parameter variation, we set the initial price to 1, initial product quality to 2, and initial service quality and personal needs met to 1. Price is held constant, and we vary the other three parameters to examine CE evolution. Thresholds are set as follows: the central NE bounds for classification from purchased users into potential NCE/NE/PCE are −0.2 and +0.2; conversion from potential CE to PCE/NCE remains ±0.2; and mutual conversion between PCE and NCE remains ±0.4. The initial fixed number of users added per day is set to 50. These values are treated as normalized scenario inputs or structural assumptions rather than empirically estimated coefficients. The robustness analysis systematically varies the conversion rate, thresholds, initial conditions, coefficient distributions/sign rules, and simulation horizon; seeded repeated runs are used for the reported uncertainty summaries. For repeated-run uncertainty summaries, the main reporting horizon is 45 periods, while the 20-period horizon is included in the horizon-sensitivity analysis. Table 2 summarizes the reference inputs and their corresponding robustness treatments.

**Table 2:** Reference inputs and robustness treatment.

Input	Reference	Status	Robustness treatment
Potential-engagement rate	0.10	Proxy-based scenario anchor (not an empirically validated behavioral conversion rate)	0.05, 0.10, 0.20
Price	1.00	Normalized scenario input	0.5-1.5; price = 3 stress check
Product quality	2.00	Normalized scenario input	1.5-2.5; 5 x 5 price-quality design
Service quality	1.00	Normalized scenario input	0-2; Stage-4-only vs continuous timing
Personal needs met	1.00	Normalized scenario input	0-2; main paired comparisons 0.5 and 1.5
Potential NE bounds	−0.20, + 0.20	Threshold extension using reference values	half-width 0.10–0.40
Manifestation thresholds	−0.20, + 0.20	Reference threshold assumption	half-width 0.10–0.30
Transfer thresholds	−0.40, + 0.40	Reference threshold assumption	half-width 0.20–0.60
Base_Add	50	Reference initial condition	25, 50, 100
Simulation horizon	45	Reference design choice	20, 45, 60; Fig 3 also visualizes T = 30
Coefficient magnitudes	Uniform(0,1)	Reference stochastic assumption	Beta(2,2), bounded normal
Coefficient signs	quality-dependent rule	Reference sign-allocation rule	fixed and asymmetric alternatives

**Fig 3:**
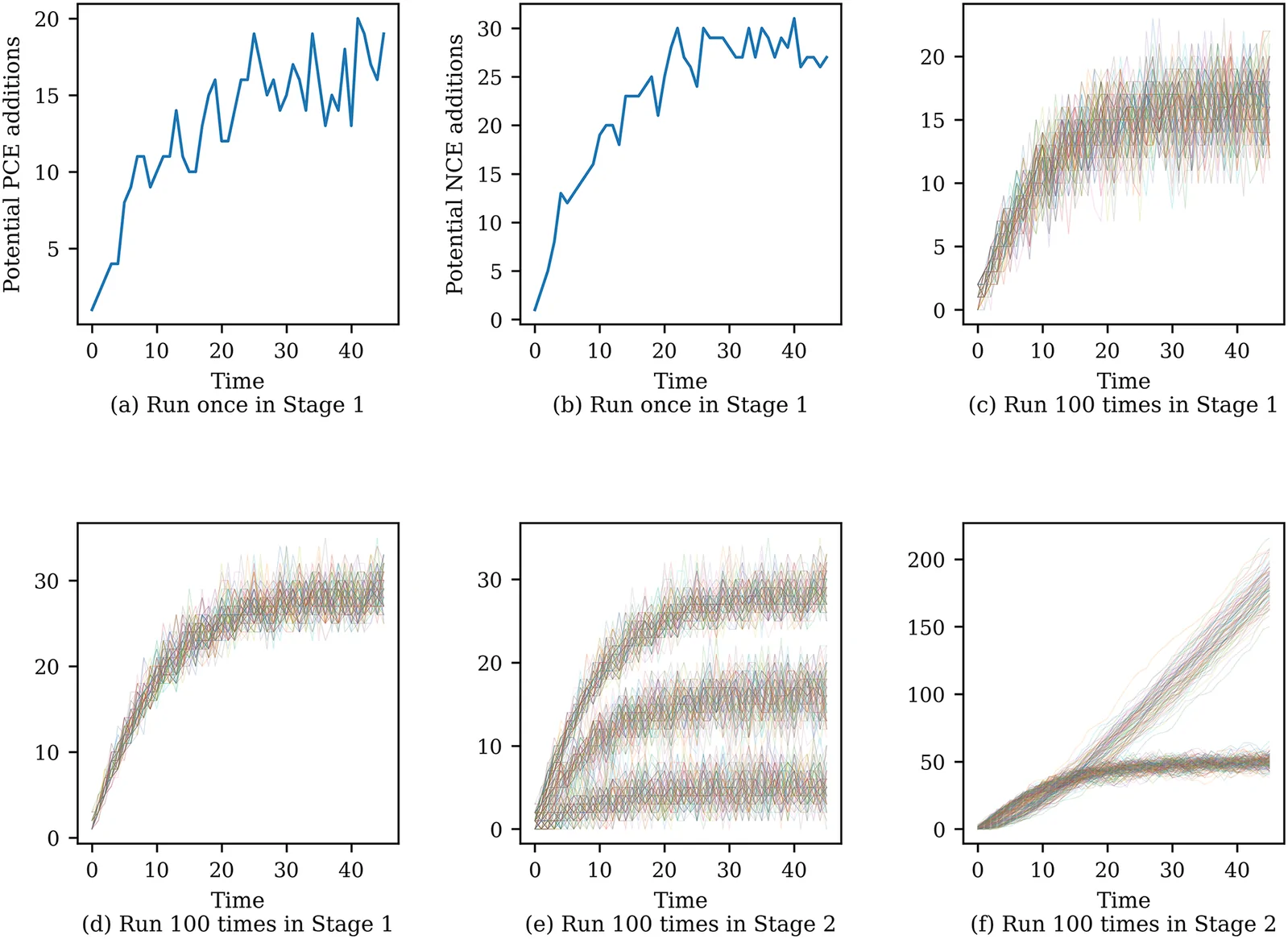
Evolution of potential PCE, NE, and potential NCE users. Panels (a) and (b) show single-run trajectories of newly added potential PCE and potential NCE users. Panels (c) and (d) show 100 representative repeated trajectories for the same quantities. Panel (e) overlays repeated trajectories for newly added potential PCE, NE, and potential NCE users, and panel (f) shows the corresponding accumulated potential-state stocks.

### 4.2 Simulation results and analysis

Based on the above parameter settings, this section presents the simulation results and analyzes the dynamic evolution of PCE and NCE users. The analysis first reports the baseline evolution of purchased users, potential engagement users, and overt engagement users across the four stages. It then examines how changes in price, product quality, service quality, and personal needs met alter the balance between PCE and NCE. This structure allows the simulation results to show not only the general evolutionary trajectory of customer engagement but also the intervention effects of different managerial factors. The analysis additionally reports repeated-run uncertainty, the explicit NE state, a direct price-by-product-quality interaction test, and systematic sensitivity and validation analyses.

#### 4.2.1 Evolution of the number of PCE, NE, and NCE users.

Fig 3 uses a six-panel potential-engagement layout that combines single-run and repeated-run views. Panels (a) and (b) show one seeded realization of newly added potential PCE and potential NCE users. Panels (c) and (d) display 100 representative repeated trajectories for the same quantities, while panels (e) and (f) incorporate NE into the repeated potential-state evolution and accumulated stocks. The panels show stochastic variation and the role of the neutral interval; the quantitative conclusions are based on 1,000 reference runs. At period 45, mean PotentialNCE is 50.33 (95% simulation uncertainty interval 45–56), mean PotentialPCE is 49.37 (41–58), and mean NE is 183.11 (162–208). The sizeable NE stock shows why a binary model can overstate polarization: many users fall inside the central evaluation interval and are therefore not forced into either potential PCE or potential NCE.

**Fig 4:**
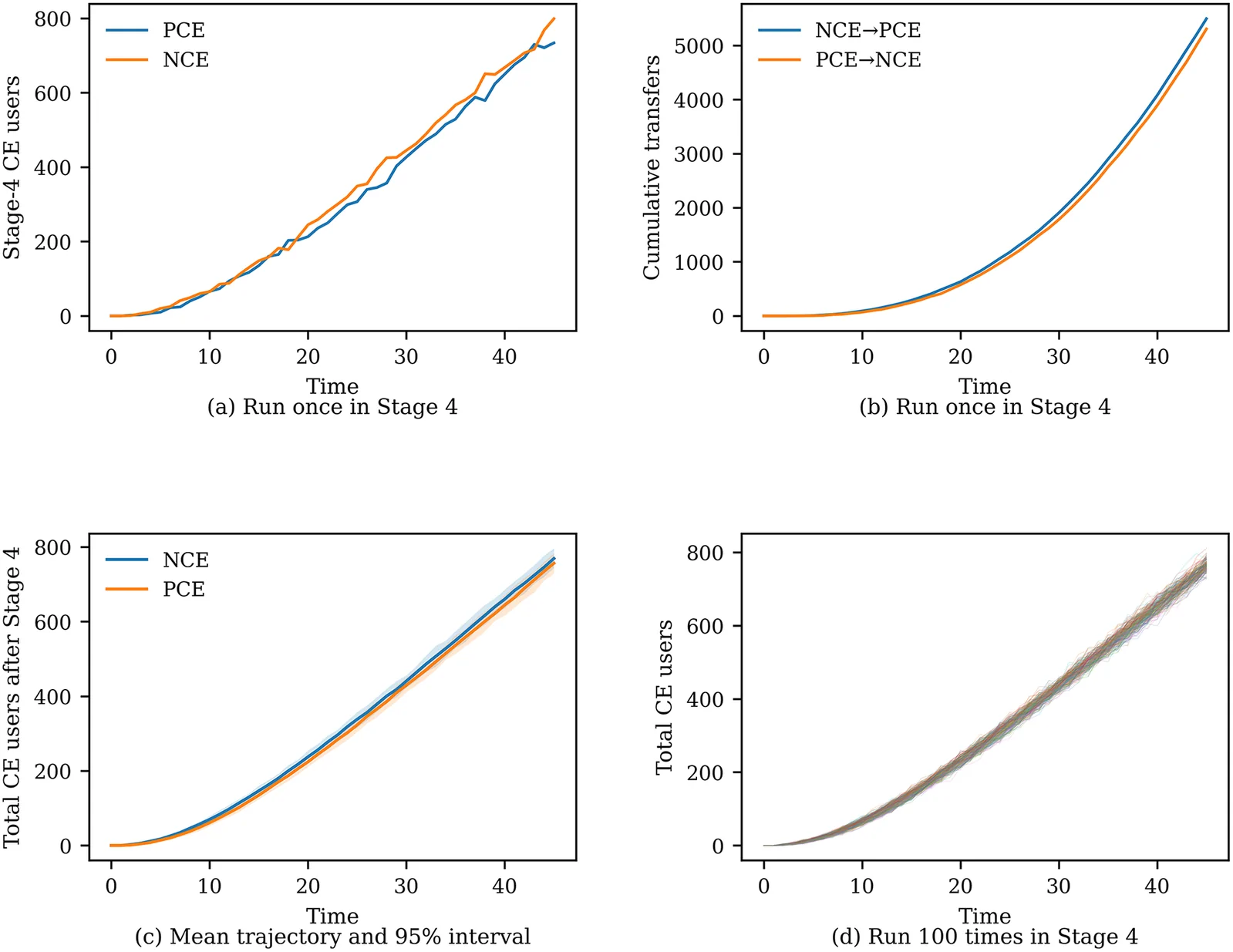
Evolution of realized PCE and NCE users after Stage 4. Panel (a) shows a single-run trajectory of realized PCE and NCE. Panel (b) shows the corresponding cumulative bidirectional transfers between NCE and PCE. Panel (c) presents repeated-run mean trajectories with 95% simulation uncertainty intervals, and panel (d) shows 100 representative repeated trajectories.

Fig 4 uses a four-panel Stage-4 layout. Panel (a) shows one seeded realization of realized PCE and NCE, panel (b) shows the corresponding bidirectional transfers, panel (c) summarizes repeated-run mean trajectories with 95% simulation uncertainty intervals, and panel (d) shows 100 representative repeated trajectories. The repeated-run evidence qualifies the interpretation of any single path. Across 1,000 reference runs at period 45, mean realized NCE is 770.11 (95% simulation uncertainty interval 744–799.03) and mean realized PCE is 755.08 (726−783). The mean PCE-NCE difference is −15.04, with a simulation uncertainty interval from −68.00 to 35.03. Thus, NCE is slightly higher on average in the reference setting, but the interval includes zero; a selected trajectory should therefore not be interpreted as a precise population prediction. NE remains outside overt PCE/NCE in the core model and is reported in Fig 5.

**Fig 5:**
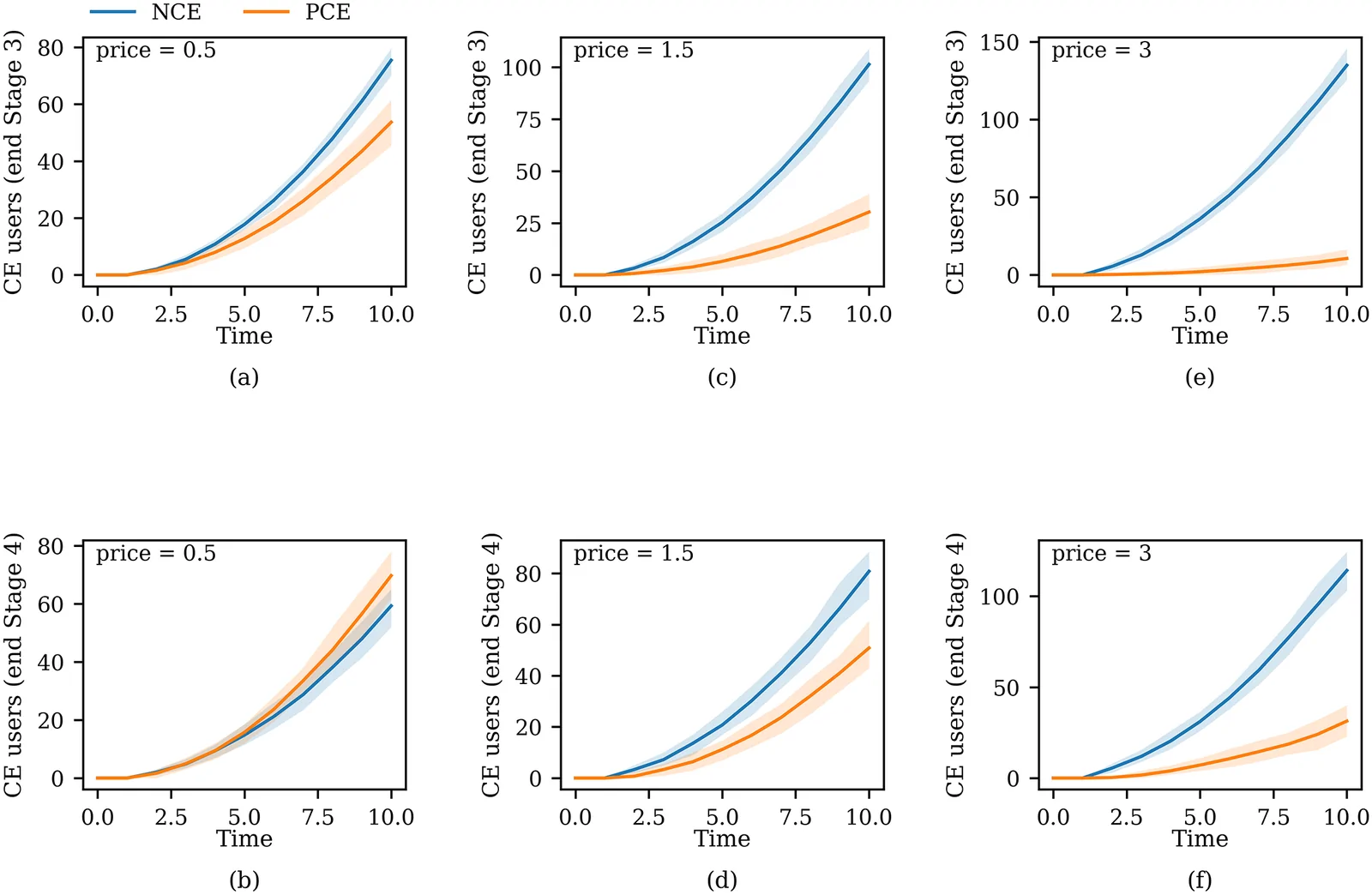
Effect of price on PCE and NCE evolution. Panels (a), (c), and (e) show repeated-run mean trajectories with 95% simulation uncertainty intervals for end-of-Stage-3 NCE and PCE under initial price settings of 0.5, 1.5, and 3, respectively. Panels (b), (d), and (f) show the corresponding end-of-Stage-4 trajectories. The price = 3 condition is an extreme stress scenario.

#### 4.2.2 The intervention effects of price on the evolution of CE behavior.

We examine price effects by varying the initial product price while holding other parameters constant. Fig 6(a)-Fig 6(d) shows PCE and NCE counts at the end of Stages 3 and 4 when price decreases from 1 to 0.5 or increases from 1 to 1.5. Although time-series patterns are similar across scenarios, price shifts the balance: lower prices reduce NCE and increase PCE, narrowing the PCE-NCE gap at both stage endpoints, whereas higher prices widen the gap. The repeated-run analysis confirms the same direction: at period 45, the mean terminal PCE-NCE balance is 224.69 when price = 0.5, −15.04 at price = 1, and −247.86 at price = 1.5; common-seed paired effects relative to the reference are + 239.73 (95% simulation uncertainty interval 166−311) and −232.83 (−312 to −155), respectively.

**Fig 6:**
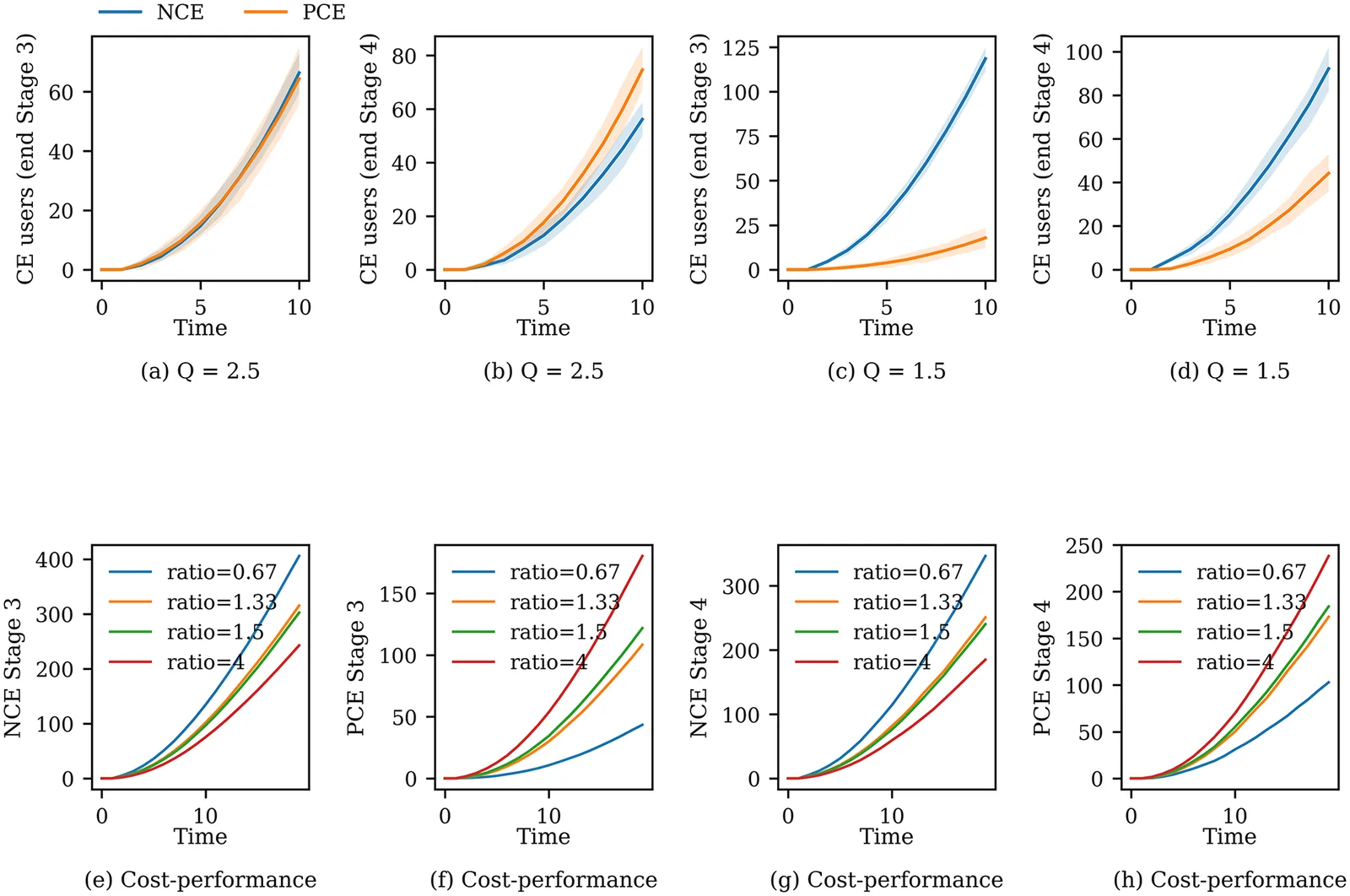
Effect of product quality on PCE and NCE evolution. Panels (a) and (b) show repeated-run end-of-Stage-3 and end-of-Stage-4 trajectories when initial product quality is 2.5; panels (c) and (d) show the corresponding trajectories when initial product quality is 1.5. Panels (e)-(h) show descriptive cost-performance scenario comparisons (0.67, 1.33, 1.5, and 4). The direct price-quality interaction test is reported separately in Section 4.2.6.

In Stage 4, bidirectional conversion occurs, but it is weaker at higher prices, leaving a large gap by the end of Stage 4. The repeated-run results preserve the directional interpretation: higher price leaves more realized NCE and fewer realized PCE users, whereas lower price produces the opposite pattern. Because price is a normalized scenario input rather than an observed willingness-to-pay measure, the numerical differences are interpreted as comparative simulation effects rather than empirical effect sizes.

The price = 3 scenario is treated only as an extreme stress check. Across 500 repeated full-horizon runs it produces a strongly negative mean terminal PCE-NCE balance (−840.49), confirming the direction of the price mechanism. Because this value lies outside the main 0.5–1.5 scenario range, it is not used as primary evidence for managerial recommendations. Fig 6 uses a six-panel arrangement: the upper row shows end-of-Stage-3 trajectories and the lower row shows end-of-Stage-4 trajectories for price = 0.5, 1.5, and 3. Each trajectory panel reports a repeated-run mean with a 95% simulation uncertainty interval rather than a selected single run. The reference price = 1 is quantified in the text rather than added as a fourth visual condition.

#### 4.2.3 The intervention effects of product quality level on the evolution of CE behavior.

We vary the initial product quality level from 2 to 1.5 and 2.5 while holding other parameters constant. Fig 7(a)-Fig 7(d) reports the resulting PCE and NCE trajectories at the end of Stage 3 and Stage 4. The effects mirror those of price manipulation. Lower product quality produces more NCE and fewer PCE users, whereas higher product quality produces fewer NCE and more PCE users. Relative to price changes, reducing product quality generates substantially higher NCE at both stage endpoints than raising price. Conversely, improving product quality yields PCE levels that are comparable to those obtained under a lower-price condition. Repeated-run evidence supports this direction: at period 45, the mean terminal PCE-NCE balance is −411.86 at quality = 1.5 and 259.66 at quality = 2.5, compared with −15.04 at the reference; the paired effects are −396.82 (−469 to −324) and +274.70 (210–343), respectively.

**Fig 7:**
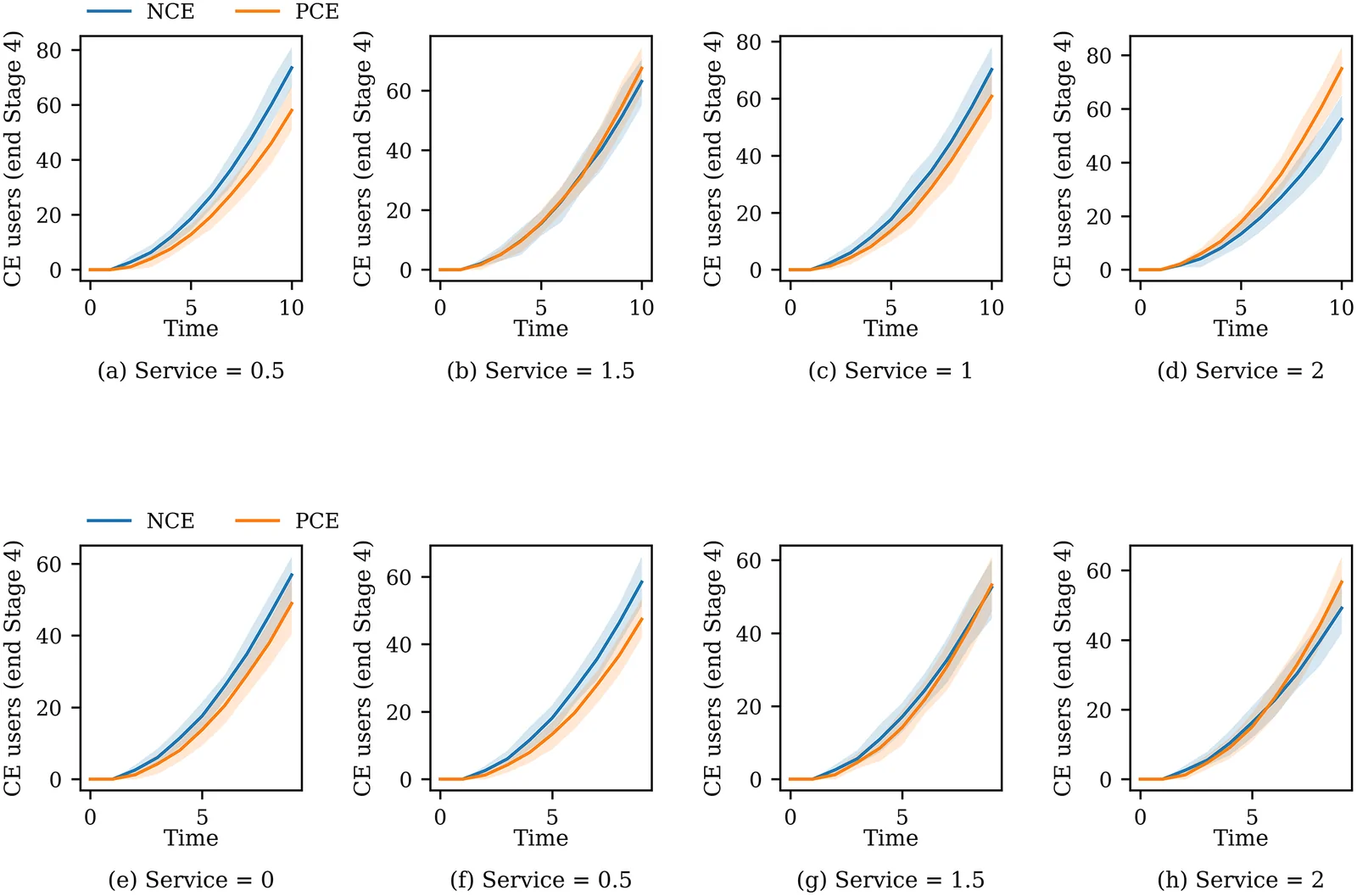
Effect of service quality on PCE and NCE evolution. Panels (a)-(d) show repeated-run trajectories under a continuous-service architecture for four service-quality settings. Panels (e)-(h) show the corresponding Stage-4-only service scenarios. Targeted recovery for NCE users is quantified separately in the accompanying text and represented conceptually in Fig 2.

Because perceived value is jointly determined by price and product quality, we also examine descriptive value-for-money scenarios based on product quality divided by price. Fig 6(e)-Fig 6(h) shows that higher descriptive value for money is associated with more PCE and fewer NCE users at the end of both Stage 3 and Stage 4. These ratio panels are treated only as descriptive analyses and are not used as direct evidence of interaction; a separate 5 x 5 price-by-product-quality factorial analysis is reported in Section 4.2.6 and Fig 8.

**Fig 8:**
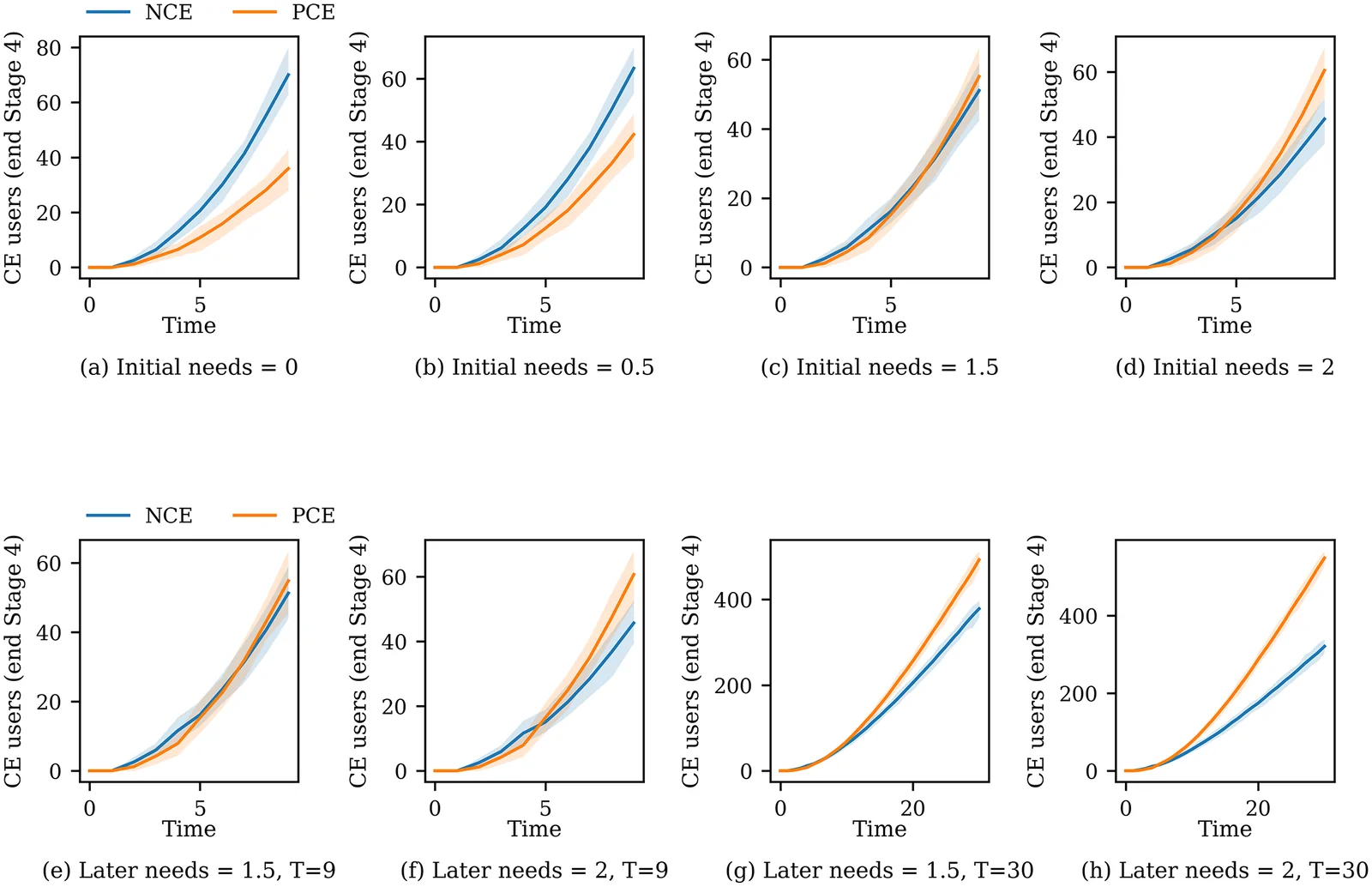
Effect of personal needs met on PCE and NCE evolution. Panels (a)-(d) compare repeated-run trajectories under four initial personal-needs settings (0, 0.5, 1.5, and 2). Panels (e) and (f) show later improvements to 1.5 and 2, respectively, and panels (g) and (h) extend the visualization horizon to 30 periods to assess persistence of the later-stage effect.

#### 4.2.4 The intervention effects of service quality level on the evolution of CE behavior.

The reference architecture makes routine service quality operative in Stage 4. Because this timing could affect the apparent service effect, we also test an alternative continuous-service architecture in which routine service quality is active from Stage 2 onward. The baseline difference between the continuous and Stage-4-only architectures is small: the paired change in terminal PCE-NCE balance is 1.41 users (95% simulation uncertainty interval −73.05 to 79.03), with favorable effects in 50.4% of runs. Thus, the timing specification alone does not create a stable difference in the baseline balance.

We then separate routine service improvement from targeted recovery. Raising service quality from 1 to 1.5 improves the terminal PCE-NCE balance by 136.01 users (63.98–207.03) under the Stage-4-only architecture and by 141.06 (65.00–216.03) when service is active continuously. The similar effects do not support a general conclusion that later-stage service investment is inherently superior. Fig 9 uses an eight-panel trajectory structure: the upper row shows continuous-service alternatives and the lower row shows Stage-4-only timing, making the architecture comparison visible within the same presentation format. Targeted recovery is evaluated as a separate paired intervention: when the same service-quality increase is applied only to NCE users, the terminal balance improves by 149.87 (76–224) and cumulative NCE-to-PCE conversion increases by 594.80 (436–731); both paired effects are positive in 100% of 1,000 runs. These results support targeted recovery rather than general later-stage superiority.

**Fig 9:**
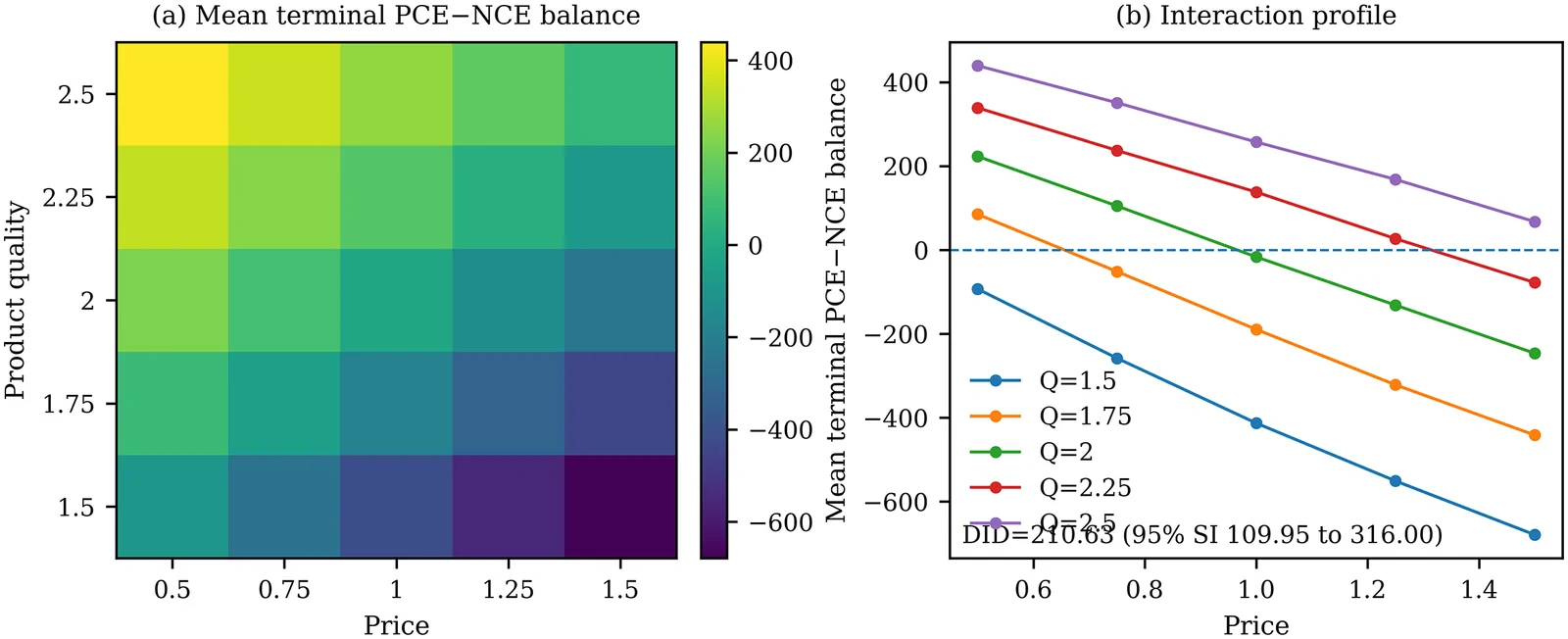
Price-quality interaction and terminal CE balance. Panel (a) presents the mean terminal PCE-NCE balance across the 5 x 5 price-by-product-quality grid. Panel (b) shows the interaction profile at each quality level. The reported difference-in-differences uses common-seed paired runs at the four corner conditions; positive values indicate that higher product quality attenuates the adverse effect of higher price.

#### 4.2.5 The intervention effects of personal needs met level on the evolution of CE behavior.

We examine the personal-needs intervention by varying personal needs met from the reference value 1 to 0.5 and 1.5. At period 45, the mean terminal PCE-NCE balance is −242.30 at 0.5 and 210.64 at 1.5. Relative to the reference, the paired effects are −227.26 (−294.03 to −154.98) and +225.68 (144.93–300.00), respectively. The repeated-run analysis therefore indicates a meaningful later-stage effect of personal needs met under the specified stage hierarchy.

Because personal needs met is activated in Stage 4 by construction, the simulation supports a later-stage association within the current hierarchy, but it cannot establish that personal needs are unimportant earlier in the customer journey. Fig 3 uses an eight-panel layout: panels (a)-(d) compare four initial personal-needs settings, panels (e) and (f) show later improvements, and panels (g) and (h) extend the visualization horizon to 30 periods to examine whether the effect persists over time. The full sensitivity analysis examines 20, 45, and 60 periods; the 30-period setting is used in Fig 3 as an additional visualization rather than as one of the archived S1 horizon-sensitivity scenarios. These checks show that horizon changes absolute state counts more than the direction of the engagement balance. We therefore avoid interpreting personal-needs interventions as inherently slower or less important than service recovery.

#### 4.2.6 Price-quality interaction analysis.

Because perceived value is jointly influenced by price and product quality [10], the descriptive quality/price scenarios are supplemented with a direct factorial test. A 5 x 5 design spans price 0.5–1.5 and product quality 1.5–2.5. The heatmap and interaction profiles in Fig 8 show that increasing quality shifts the terminal PCE-NCE balance toward PCE at each tested price, whereas increasing price shifts it toward NCE at each tested quality. Using the four corner conditions with common random seeds, the difference-in-differences in terminal PCE-NCE balance is 210.63 (95% simulation uncertainty interval 109.95–316.00), and all 500 paired runs are positive. Thus, higher quality attenuates the negative effect of higher price, but the appropriate interpretation is an interaction effect rather than a universal quality/price-ratio rule.

### 4.3 Sensitivity, uncertainty, and validation

Systematic sensitivity analysis varied the potential-engagement rate, the new NE interval, manifestation and transfer thresholds, Base_Add, the simulation horizon, coefficient-magnitude distributions, and coefficient sign-allocation rules. Potential-rate and horizon changes primarily affect population scale, while the normalized PCE-NCE balance remains comparatively stable. Widening the potential neutral interval predictably increases NE. Results are also similar when Uniform coefficient magnitudes are replaced by Beta(2,2) or bounded normal alternatives. S1 Fig summarizes the sensitivity ranges and the sign-allocation robustness checks; the complete scenario-level outputs are included in the reproducibility package.

The principal structural uncertainty is coefficient sign allocation. Under the reference quality-dependent sign rule, the terminal PCE-NCE balance is close to zero on average. Fixed or asymmetric negative-sign probabilities can shift the balance substantially toward PCE or NCE. The Assignment_Perception_Coefficient rule is therefore treated as a structural assumption rather than an empirically estimated distribution. Main managerial conclusions are framed around paired directional effects of price, quality, needs, and targeted recovery rather than around the exact absolute share of PCE and NCE. The reference sign rule has no independently established theoretical or empirical distribution, so the tested alternatives cannot be used to select a population-predictive specification. Accordingly, all substantive conclusions are limited to directional comparisons that remain robust across the reported tests, not forecasts of actual state shares.

Internal validation included seed reproducibility, exact population conservation, non-negative states, zero-conversion boundary conditions, neutral-band behavior, directional extreme checks, and service-architecture conservation; all 12 pre-specified checks passed. Empirical validation is intentionally modest. Previously coded Xiaomi evidence contains more negative than positive reviews (537 versus 326), documents users who display both positive and negative engagement over time, and identifies service satisfaction and personal-needs fulfillment among prominent antecedents of NCE-to-PCE change. These patterns are directionally consistent with the simulation but are not used to claim point calibration or exact prediction of community state shares. Table 3 summarizes the sensitivity and validation findings.

**Table 3:** Summary of sensitivity and validation findings.

Domain	Alternative settings	Main conclusion
Potential-engagement rate	0.05-0.20	Mainly changes scale; normalized polarity comparatively stable
Potential NE half-width	0.10-0.40	Wider interval increases NE as expected
Manifestation threshold half-width	0.10-0.30	Direction of reference balance remains similar
Transfer threshold half-width	0.20-0.60	Moderate changes in transfer frequency; reference direction stable
Horizon	20, 45, 60	Absolute counts change; direction of normalized balance is similar
Base_Add	25, 50, 100	Changes population scale
Coefficient magnitude distribution	Uniform, Beta(2,2), bounded normal	Reference direction broadly similar
Coefficient sign allocation	Reference rule plus fixed/asymmetric alternatives	Major structural uncertainty; absolute PCE/NCE balance can reverse
Internal validation	12 pre-specified checks	All passed
Empirical pattern validation	Coded Xiaomi summaries	Directional/structural support; not point calibration

## 5. Discussion

The four-stage simulation yields four substantive patterns: a sizeable non-engagement stock remains between positive and negative manifestations; price and product quality operate jointly through perceived value; routine service quality and targeted recovery are not equivalent interventions; and personal-needs fulfillment changes later outcomes within the imposed stage sequence. These findings are interpreted as robust directional comparisons across tested scenarios, not as forecasts of the actual proportions of PCE, NCE, or NE users in Xiaomi Community or another population.

The NE result is theoretically important because prior CE scholarship defines engagement as customers’ cognitive, affective, and behavioral investment in a focal brand and recognizes that this investment can have positive, negative, or dual valence [1–7]. Those concepts do not imply that every antecedent evaluation must become an observable advocacy or complaint behavior. In the present model, NE is therefore not proposed as a new engagement construct. It is a non-manifestation stock that operationalizes the possibility that users evaluate the brand without crossing either behavioral threshold. Making this stock explicit prevents a binary simulation from inflating polarization and permits population accounting in which potential NCE, NE, potential PCE, realized NCE, and realized PCE remain distinct.

The price and product-quality results are consistent with perceived-value logic, in which price represents sacrifice and quality represents received benefits [10]. Their positive interaction means that a quality improvement attenuates the adverse directional effect of a price increase within the tested normalized range. In a technology-oriented OBC, this relationship is plausible because community members discuss not only the purchase price but also product performance, reliability, software support, and the consequences of product problems [8,9]. The result does not estimate a willingness-to-pay elasticity or validate a universal quality-price ratio. Instead, it shows why price and product decisions should be interpreted as a joint value proposition: a price increase is more likely to generate negative engagement when users cannot identify a commensurate improvement in product benefits.

The service findings require a distinction between routine service quality and service recovery. Routine service quality is a broad evaluation of ordinary support across the customer journey [11]. Service recovery is event-contingent: it follows a failure or unresolved problem and seeks to restore the affected customer's evaluation and relationship [12,13]. The architecture comparison shows that general service improvement changes the PCE-NCE balance similarly whether routine service enters continuously or only in Stage 4, so the model does not support a general proposition that later service is superior. By contrast, targeted recovery is applied only to users already in NCE and directly tests whether corrective action can move that stock toward PCE. The narrower recovery conclusion is therefore more defensible because the intervention, eligible population, and modeled transition correspond to the same failure-repair mechanism.

The personal-needs result is compatible with research that treats customer motives and fit as antecedents of engagement [14–16], but its timing is conditional on the model. Because personal needs met is activated in Stage 4 by construction, the simulation shows what follows under a later-stage fit mechanism; it cannot establish that needs are absent or weaker earlier in the journey. This distinction illustrates a broader interpretive rule for stage-based simulation: the model can compare consequences of a specified temporal ordering, whereas empirical longitudinal data are required to establish that the ordering describes actual customer behavior.

Finally, repeated runs and sensitivity tests shift the role of the model from single-trajectory demonstration to transparent mechanism exploration. Directional effects of price, quality, needs, and targeted recovery persist across the reported paired comparisons, while absolute state balances vary materially with coefficient sign allocation. Consequently, the contribution lies in exposing how assumptions generate transitions and in identifying comparisons that survive the tested alternatives. It does not lie in predicting the number or share of advocates, detractors, or non-engaged users in a real community.

## 6. Conclusions

Building on the simulation results, this section discusses theoretical and managerial implications in light of the repeated-run, interaction, architecture, and sensitivity analyses. The four-stage process remains the organizing framework, while the conclusions distinguish robust directional mechanisms from outcomes that depend more heavily on structural assumptions.

### 6.1 Theoretical Implications

First, the model connects the dynamic and dual-valence view of CE [1,2,7] with an explicit non-manifestation state. Prior literature establishes that engagement may be positive, negative, or ambivalent, but this does not require every brand evaluation to produce observable engagement. The theoretical contribution is therefore not the discovery that some customers remain inactive. It is the formal integration of that possibility into a stage-based CE process, where NE is mutually exclusive from potential and realized PCE/NCE and where its inclusion prevents moderate evaluations from being mechanically polarized.

Second, separating antecedent evaluation from engagement is treated as a construct-validity requirement rather than an independent theoretical contribution. CE theory already distinguishes the focal engagement state or behavior from the appraisals and motives that precede it [3–6,14–16]. The model's added value is to enforce this distinction throughout the transition system: price, product quality, service quality, and personal needs met affect latent evaluation, thresholds map that evaluation into engagement states, and the resulting stocks represent manifestations rather than the antecedents themselves. This makes the computational mechanism auditable against the conceptual domain of CE.

Third, the service analysis refines the temporal interpretation of engagement repair. Routine service quality can influence evaluations before or after a negative episode, whereas targeted recovery is conditional on a failure and on the presence of an affected customer [11–13]. The finding that routine-service timing produces similar directional effects removes support for a broad later-stage-superiority claim. The stronger theoretical statement is narrower: when negative engagement has already manifested, a failure-contingent corrective mechanism is aligned with the NCE-to-PCE transition and is more defensible than attributing the change to general service timing. Viewed at a broader business-model level, this recovery logic is also consistent with resilience perspectives that emphasize adaptability and the capacity to preserve and renew value creation under disruption [20].

Fourth, the uncertainty analysis clarifies what simulation can contribute to CE theory. By separating proxy-based scenario anchors, normalized inputs, and structural rules, the study shows which conclusions follow from the mechanism and which depend on parameterization. Robust directional comparisons can inform propositions for subsequent empirical testing; absolute PCE, NCE, and NE shares cannot. This boundary turns structural uncertainty into an explicit part of theory development rather than concealing it behind a single trajectory.

### 6.2 Managerial Implications

The price-quality interaction suggests that firms should manage a price change as a value-communication program rather than as an isolated number. For a Xiaomi-style smartphone community, an illustrative program would link each price increase to verifiable benefits such as improved battery durability, camera performance, repairability, software-support duration, or warranty coverage. A side-by-side upgrade matrix, community demonstrations, early-user trials, and transparent explanations of which user problems the added features solve can make the quality increment visible. This is an implementation illustration, not a claim that Xiaomi currently uses these specific practices.

Managers can also segment the response instead of assuming one message will fit all users. Price-sensitive users may value trade-in support, installment options, or a lower-priced configuration, whereas performance-oriented users may respond to benchmark evidence and longer support commitments. Community monitoring should then compare issue-specific complaint volume, unresolved-case recurrence, and the direction of subsequent user contributions before and after the change. These indicators can reveal whether the communicated quality gain is actually offsetting the negative value appraisal; the simulation itself does not supply a numerical price premium or target ratio.

For established NCE, the results support a targeted recovery protocol. A practical sequence is to identify a concrete negative episode, classify whether it concerns hardware, software, delivery, or after-sales service, assign a case owner, provide a remedy appropriate to that cause, and follow up after resolution. In a smartphone community, remedies could include diagnostic support, a repair or replacement path, a software rollback or fix timetable, an extended service commitment, and a public explanation when the issue affects many users. Inviting the customer to confirm whether the problem was resolved closes the recovery loop; pressuring the user to post positively would not. Routine service should continue for all users, but recovery resources should be concentrated where a documented failure and NCE signal coincide. More broadly, recurring NCE and recovery outcomes can be treated as feedback for adapting service routines and value communication, embedding recovery capability in a resilient business model rather than treating each negative episode as an isolated response [20].

Personal-needs fit can be managed through product-function guidance, accessibility and ecosystem support, and community content organized around recognizable use cases. Managers should treat the model as a decision aid for prioritizing the direction and timing of interventions, then validate each intervention with observed retention, resolution, and engagement measures. Because the inputs are normalized and the sign structure is not empirically calibrated, none of the simulated counts should be used as staffing forecasts, market-share estimates, or numerical performance targets.

### 6.3 Limitations

The potential-engagement rate of 0.10 is only a proxy-based scenario anchor derived from descriptive membership and sales quantities. Community membership, product purchase, and potential engagement are related but non-equivalent, so 0.10 is not an empirically validated behavioral conversion rate or calibrated probability. The tested scenario outputs are therefore used for robust directional comparisons and are not interpreted as predictions of actual PCE, NCE, or NE population shares.

The study does not conduct a record-level parameter-calibration exercise and cannot report a formal goodness-of-fit statistic against longitudinal community state transitions. Empirical support is limited to directional consistency checks using privacy-preserving aggregate Xiaomi patterns. In addition, the coefficient sign-allocation rule has no independent theoretical derivation or empirical estimate and is the most consequential structural assumption; alternative sign rules can reverse the absolute PCE-NCE balance. Repeated runs, internal conservation checks, and sensitivity analyses improve transparency and assess robustness, but they do not substitute for external calibration or predictive validation.

Future research should estimate conversion rates, thresholds, coefficient distributions, and sign structures from de-identified longitudinal OBC data; reserve a holdout period or community for out-of-sample validation; and compare simulated and observed transition matrices with formal fit criteria. It should also examine whether NE users re-enter polarized engagement states, whether personal needs operate before Stage 4, and whether the mechanism replicates across product categories and communities. Nonlinear evaluation, learning, network ties, opinion leaders, and emotional contagion are valuable extensions, but each requires independent theoretical justification and empirical identification.

## Supporting information

S1 Fig Robustness and sensitivity summary. Panel (a) summarizes the range of normalized terminal PCE-NCE balance across the tested parameter and structural alternatives. Panel (b) compares the coefficient sign-allocation rules, which constitute an important structural source of variation in the absolute PCE-NCE balance.
(TIF)
